# Heater-Associated Erythema Ab Igne: Case Report and Review of Thermal-Related Skin Conditions

**DOI:** 10.7759/cureus.8057

**Published:** 2020-05-11

**Authors:** Parnia Forouzan, Ryan R Riahi, Philip R Cohen

**Affiliations:** 1 Dermatology, University of Texas Medical School, Houston, USA; 2 Dermatology, DermSurgery Associates, Sugar Land, USA; 3 Dermatology, San Diego Family Dermatology, National City, USA

**Keywords:** ab, carcinoma, erythema, heat, heater, igne, skin, thermal, ultraviolet, urticaria

## Abstract

Erythema ab igne is a thermal-associated skin condition that can occur secondary to persistent direct or indirect contact with heat. Historically, erythema ab igne has been linked to fireplace and stove exposures; more recently, it has been associated with heaters, hot water bottles, and laptops. A 48-year-old woman presented for the evaluation of hyperpigmented, reticulated macular lesions on her distal legs. Additional history revealed that she had developed erythema ab igne secondary to the use of a space heater underneath her desk at work. Her skin condition stopped progressing with removal of the causative agent. In addition to erythema ab igne, heat-related skin conditions include basal cell carcinomas and squamous cell carcinomas, burns, erythromelalgia, subtypes of urticaria, and ultraviolet-associated disorders. Awareness of thermal-associated skin conditions enables the clinician to establish the appropriate diagnosis based on the associated history of the condition, the morphology of the skin lesion, and, if necessary, correlation with the skin biopsy findings of the cutaneous condition.

## Introduction

Erythema ab igne is an unintentional thermal-associated adverse cutaneous disorder that can occur following repeated exposure to an exogenous heat source. Initially, this skin condition presents as net-like, erythematous bands that become darker and fixed with persistent exposure to the causative agent. Common heat sources include fireplaces, heating pads, hot water bottles, laptops, and space heaters [1,2].

In addition to erythema ab igne, other disorders can be classified as thermal-mediated skin conditions. These include basal cell carcinomas and squamous cell carcinomas, certain subtypes of urticaria, and miscellaneous conditions that can affect the skin, such as burns, erythromelalgia, and ultraviolet-mediated skin disorders. These injuries may occur as a result of direct or indirect exposure to the causative heat factor.

A woman who developed erythema ab igne as a result of repeated exposure to a space heater is described. In addition, the literature has been surveyed, and a comprehensive list of thermal-associated skin conditions is reviewed.

## Case presentation

A 48-year-old woman presented for the evaluation of an itchy darkening of the skin on her lower legs. She noticed that the lesions initially appeared one year earlier. She had no changes to her medications.

**Figure 1:**
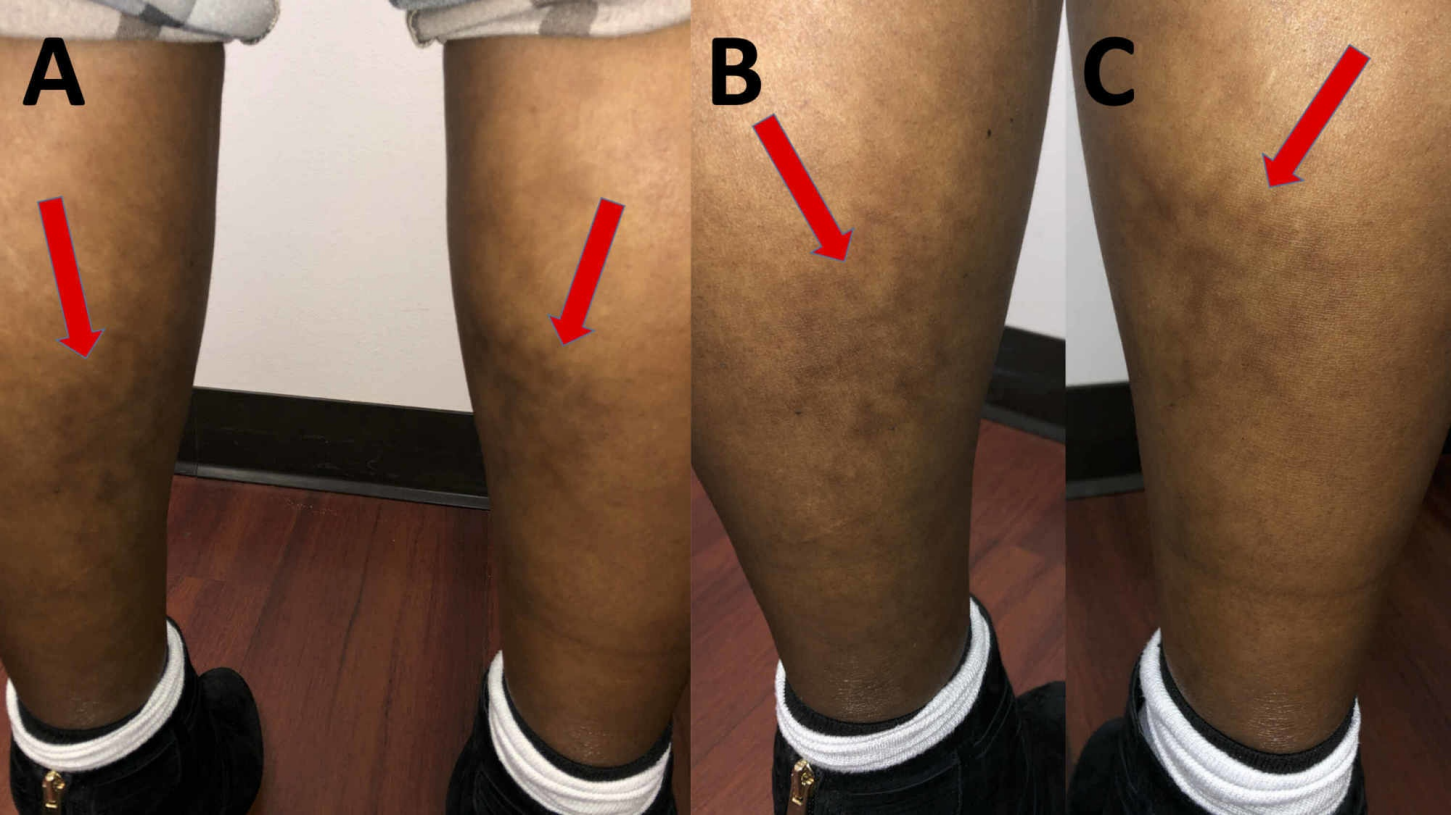
Clinical presentation of heater-associated erythema ab igne on the legs of a 48-year-old woman Distant (A) and closer (B and C) posterior view of the posterior distal left leg (B) and right leg (C). Erythema ab igne clinically appears as hyperpigmented, reticulated bands (red arrows).

Cutaneous examination revealed a woman with Fitzpatrick skin type IV; her skin color was moderate brown, and she minimally burned and always tanned well after sun exposure. She had hyperpigmented, reticulated patches on the anterior and posterior surfaces of both lower legs (Figure 1).

Additional history, requested after evaluating her legs, revealed that she used a space heater under her metal desk at work because she was always cold in her office. Correlation of the patient’s history and the clinical morphology of her skin lesions established a diagnosis of erythema ab igne. She was advised to immediately discontinue the use of the space heater at work.

## Discussion

Thermal-associated skin conditions may result from direct (heat source contacting the skin) or indirect (heat source in close proximity to but not contacting the skin) exposures to heat. These disorders can be classified by either their presentation, source of heat, or both: carcinomas, ultraviolet-associated skin disorders, urticaria, and miscellaneous conditions, including angioedema, burns, erythema ab igne, and erythromelalgia (Table 1) [1-20].

**Table 1:** Thermal-associated skin conditions

Skin conditions		References
Carcinomas	Basal cell carcinoma	[3-5]
	Squamous cell carcinoma	[6-8]
Ultraviolet-associated skin disorders	Beach feet	[9]
	Sunburns	[10]
Urticaria	Cholinergic urticaria	[11,12]
	Localized heat urticaria	[13]
	Solar urticaria	[14]
Miscellaneous	Angioedema	[15]
	Burns (first-degree, second-degree, and third-degree)	[16-19]
	Erythema ab igne	[1,2]
	Erythromelalgia	[20]

Specific clinical features, pathology findings, and associated history aid in the diagnosis of thermal-associated conditions. The salient features of these conditions are reviewed. In addition, clinical examples of patients with thermal-associated skin conditions are summarized.

Although nonmelanoma skin cancer is typically associated with ultraviolet radiation, basal cell carcinoma and squamous cell carcinoma can also rarely occur secondary to thermal injury. Heat-induced basal cell carcinomas account for less than one percent of all basal cell carcinomas, and basal cell carcinomas make up 12 percent of tumors that develop on burn scars. Treatment for these malignancies often requires excision of the tumor [3-5].

The morphology of basal cell carcinomas is variable; it ranges from a flesh-colored papule to a pink, raised, shiny plaque [3-5]. Pathology shows aggregates of basaloid tumor cells with hyperchromatic and large nuclei, minimal cytoplasm, and peripheral palisading. Basal cell carcinomas can result from previous burns or the use of rimless glasses or heated lamps [3-5].

An 80-year-old woman presented with a pearly, pink plaque on her left vulva. Microscopic examination established the diagnosis of vulvar basal cell carcinoma. Her history revealed repeated exposures to perineal heat lamps primarily used by postpartum patients for healing and pain relief. She had received twice daily treatment with the lamp after each of her five pregnancies. The repeated infrared radiation exposure was postulated to be the cause of her vulvar basal cell carcinoma [3].

Basal cell carcinoma has also been hypothesized to result from the chronic use of rimless spectacles; the refracted light elevates the skin temperature by an additional three degrees Fahrenheit in one minute where light is focused. Persistent use of such eyeglasses can lead to cutaneous changes on the skin below the lower edge of the lenses. Twelve patients presented with thermal-induced cutaneous changes ranging from erythematous, scaly growths to telangiectatic, translucent papules resulting from use of rimless glasses; biopsy revealed basal cell carcinoma in four patients [4].

Burn scars can also lead to the development of skin cancer: most commonly squamous cell carcinomas but also basal cell carcinomas. A 55-year-old man had a 50-year-old scar from a boiling water burn. He subsequently presented with scaly lesions that were limited to the burn scar surface. Biopsy revealed basal cell carcinoma [5].

Squamous cell carcinomas can present as erythematous, scaly plaques with a central ulceration; pathology ranges from well-differentiated to poorly differentiated tumor cells. Marjolin ulcers are aggressive skin cancers that develop in previously burned skin, old scars, and damaged skin. They present with induration and nodules and are often associated with the development of squamous skin carcinomas. A 51-year-old man presented with a firm, ulcerated mass and nodular growths that had appeared three years prior on a childhood burn scar; a biopsy revealed a squamous cell carcinoma which was excised [6].

Erythema ab igne can be associated with the appearance of a squamous cell carcinoma in the lesion. A 60-year-old woman had a 35-year habitual exposure to a fire stove in the winter. She presented with a hyperpigmented, net-like erythema ab igne on her lower legs in addition to hyperkeratotic, well-demarcated plaques and nodules of squamous cell carcinoma. Her chronic exposure to exogenous heat resulted in the development of the squamous cell carcinoma within the area of erythema ab igne [7].

Kangri cancer is a type of squamous cell carcinoma. It has been described in patients from Kashmir. Kangri cancer has been linked to using a Kangri fire pot as a heat source in the winter [8].

The Kairo cancer of Japan and the Kang cancer of China are also squamous cell carcinomas associated with body warming techniques, such as sleeping on hot bricks and carrying burning flasks. These skin cancers occur at sites where the skin is in contact with these warming devices. Indeed, the pathogenesis of these skin cancers is similar to the cutaneous neoplasms developing in erythema ab igne [7,8].

Ultraviolet radiation is present in sunlight and includes wavelengths sized between 10 and 400 nanometers. Ultraviolet radiation can directly damage the skin resulting in premalignant (actinic keratosis) and malignant (skin cancer) conditions. This radiation may also induce thermal injury through contact with ultraviolet-mediated hot surfaces [9,10].

Beach feet is an ultraviolet radiation-associated thermal condition. It presents as erythematous patches with blisters on the plantar surface of the feet and toes after running barefoot on sunlight-induced hot sand. The ultraviolet radiation-induced hot sand surface transfers large amounts of heat to the runner’s feet. Subsequently, tender, red areas with blister formation occur on the soles of the individual’s feet [9].

Sunburn is another thermal injury that can result from exposure to ultraviolet radiation. The ultraviolet index is a measurement, at a specific place and time, that reflects the strength of ultraviolet radiation present to produce a sunburn. It is higher on hot, sunny days. In addition, prolonged exposure time to ultraviolet radiation can increase the risk and severity of sunburns. Similar to sunburns, the use of tanning beds is associated with ultraviolet radiation-induced skin burns [10].

Sunburns appear as diffusely erythematous, tender skin in locations that have been exposed to solar radiation. Sunburns are typically classified as first-degree burns. However, second-degree burns (characterized by bullae and deeper damage) are possible from sunburns [10].

An 11-year-old boy presented with first- and second-degree burns following repeated hot air balloon rides. He presented with blisters, edema, and erythematous patches covering his back, forearms, and shoulders. Treatment for sunburns may include nonsteroidal anti-inflammatory drugs and symptomatic relief with aloe vera gel, menthol, and other topical cooling agents [10].

Urticaria, or hives, present as pruritic, erythematous, raised areas of skin. They are commonly referred to as wheals. The individual lesions typically resolve within 24 hours [11].

Urticaria can be triggered by physical or nonphysical etiologies. Microscopic examination shows edema in the dermis and a sparse perivascular infiltrate of lymphocytes and often eosinophils. Types of thermal-induced urticaria include cholinergic, localized heat, and solar urticaria [11].

Cholinergic urticaria is thought to be mediated by acetylcholine. It can be induced by emotional stress, exercise, and spicy foods. However, cholinergic urticaria can also be thermal related in etiology [11].

The skin lesions in cholinergic urticaria are often smaller; they can be localized or generalized in distribution. Similar to cholinergic urticaria, cholinergic pruritus is associated with the same triggers but only presents with pruritus instead of raised urticarial lesions [11].

A 39-year-old woman presented with localized, recurrent pruritic plaques on her torso that were triggered by mild heat and exercise. Cutaneous examination revealed small wheals and excoriation without dermatographism. She was diagnosed to have cholinergic urticaria. She obtained some relief by applying ice packs to the symptomatic areas and by keeping her bedroom cool. In addition, treatment with omalizumab was successful in decreasing her episodes of heat-associated cholinergic urticaria [12].

Localized heat urticaria can be triggered by exposure to heat such as contact with hot water with a mean instigating temperature of 45 degrees Celsius. In contrast to cholinergic urticaria, it is not caused by exertion or sweating. Also, in contrast to solar urticaria, heat urticaria is solely temperature dependent [13].

A 38-year-old woman presented with recurrent erythematous, well-demarcated wheals that would resolve 40 minutes after the subjection to heat. The diagnosis of localized heat urticaria was confirmed with heat provocation testing which revealed erythematous, itchy wheals five minutes after exposure to hot water or sunlight. Similar to other forms of urticaria, localized heat urticaria can be treated with antihistamines or, if refractory, antibodies against immunoglobulin E such as omalizumab [13].

The final type of thermal-associated urticaria is solar related; it occurs after exposure to sunlight. A 30-year-old woman would develop edematous, pruritic, erythematous, skin wheals within 10 minutes of sun exposure to the areas. Sunscreen did not prevent her solar urticaria. However, treatment with omalizumab alleviated her symptoms [14].

There are several heat-induced cutaneous conditions that do not fit into the former categories: angioedema, burns, erythema ab igne, and erythromelalgia. Angioedema demonstrates edema of the deep dermis. It is often associated with allergic reactions and certain medications.

A 20-year-old woman experienced recurrent swelling of her eyelids with exposure to hot water greater than 41 degrees Celsius. She did not develop urticaria with each episode; however, she did experience respiratory discomfort with one episode and itchy eyelids with every episode. Her symptoms could be abated with prior antihistamine treatment or would spontaneously resolve [15].

Burns can be precipitated by agents such as chemicals, friction, heat, and radiation, each with a different pathophysiological response. The burn depth (first-degree, second-degree, and third-degree) can help categorize the injury and appropriate treatment (Table 2) [16-18]. In addition, burns may lead to later development of skin malignancies [5,6,16].

**Table 2:** Comparison of features of first-degree, second-degree, and third-degree burns

Type of burn	Symptoms	Histology	Scarring potential	Treatment	References
First-degree	Red and painful skin	Epidermis begins to separate from the dermis, enlarged nuclei in epidermal cells, and dilated vessels in dermis	Usually no scarring	Resolve spontaneously without the need for medical treatment	[16,17]
Superficial second-degree	Red, painful, and may lead to blistering of skin	Detachment of epidermis from dermis and cytoplasmic vacuoles in basal cells	May scar	Resolves without the need for medical treatment	[16,17]
Deep second-degree	Pale, less painful, and may lead to blistering of skin	Similar to superficial second-degree burns	Often scars	Typically requires surgery and may also need antibiotics	[16-18]
Third-degree	Dry, leathery, darkened skin but not painful	Coagulative necrosis of dermis and epidermis	Will scar	Requires surgery but may also need antibiotics and fluids	[16,17]

First-degree burns only affect the epidermis. They cause redness and pain that is limited in duration. Microscopic examination usually reveals that the epidermis is beginning to detach from the dermis. In addition, the nuclei of cells in the epidermis enlarge, and the vessels in the dermis become dilated. First-degree burns typically resolve spontaneously without scarring [16,17].

Superficial second-degree burns are more painful and lead to blistering. These burns can scar. However, they usually do not require surgery [16].

Deep second-degree burns are less painful. Yet, they clinically resemble superficial second-degree burns. Typically, they require surgery and often scar. Microscopic examination of second-degree burns shows detachment of the epidermis from the dermis and cytoplasmic vacuoles in basal cells of the epidermis [16,17].

Three patients with thermal-induced burns resulting from the combustion of aerosol sprays were described. They developed first- and second-degree burns on the head, neck, and upper extremities. The affected areas presented as pink, wet-appearing burns with interspersed bullae as a result of the flames. All of the patients recovered with little to no residual scarring after treatment with topical silvadene, bacitracin, and/or debridement [18]. 

Third-degree burns involve destruction of the full thickness of the skin. The cutaneous presentation is dry and leathery. Third-degree burns are usually not painful due to the loss of pain receptors; however, these burns do require surgery and will scar. Microscopic examination of a third-degree burn shows coagulative necrosis of the dermis and epidermis [16,17].

A three-year-old girl presented with a third-degree burn characterized by an extensive, well-demarcated necrosis of her skin following placement of a heated cryogel pack to ease swelling in her foot. Her burn was treated with debridement and a skin graft. She had no subsequent complications [19].

Erythema ab igne results from prolonged heat exposure insufficient to cause a burn. It presents as hyperpigmented, reticulated bands. Patients may also experience burning and itching [1,2].

Similar to the patient in this report, erythema ab igne is often an unintended result of obtaining warmth from an exogenous source. This skin condition may be associated with hypothyroidism. Thermal sources that have resulted in erythema ab igne include the chronic use of car seat heaters, heating blankets, heating pads, hot water bottles, laptops, radiators, and stoves [1,2].

Our patient presented with erythema ab igne on her lower legs after prolonged space heater use underneath her desk. Initial management involved the removal of the heat source. Eventually, the erythema ab igne-associated hyperpigmentation may fade. If continued exposure to the thermal source is not eliminated, nonmelanoma skin cancer may develop at the erythema ab igne site [1,2,5].

A 19-year-old woman presented with erythematous, net-like hyperpigmentation on her legs. The reticulated bands had darkened over the course of two months. Her history revealed that she worked in a cold environment and utilized a space heater underneath her desk to keep warm. A diagnosis of erythema ab igne was made, similar to our patient [2].

A 21-year-old woman developed hyperpigmentation with a reticular pattern on her thighs; however, it was notably more pigmented on her left thigh. The patient reported prolonged use of her laptop while it rested on her thighs. The areas of hyperpigmentation correlated with the laptop placement and were more pronounced under the laptop’s heating element on her left thigh. After avoiding direct exposure, the woman’s hyperpigmentation did not fade [1].

Erythema ab igne has also been reported with chronic exposure to heated blankets and heating pads. Patients in intensive care units who utilize electric blankets have been observed to develop erythema ab igne. In addition, patients using heating pads to alleviate chronic pain have been reported to exhibit this hyperpigmented band pattern [1,2].

This hyperpigmented pattern can also develop on the face and shins of cooks persistently using stoves. Similarly, elderly individuals who repeatedly sit by the fireplace can develop erythema ab igne on their exposed skin, most commonly on their lower legs [1,2].

Biopsies are usually not required to establish the diagnosis of erythema ab igne. However, the pathology changes of early erythema ab igne include the accumulation of elastic fibers and vasodilation in the dermis and increased melanin in the epidermis. In later stages, epidermal changes include hyperkeratosis and vacuolization of the cells in the stratum spinosum [1,2,7].

Erythromelalgia is a rare skin condition that presents with burning pain, edema, erythema, and elevated temperature in the extremities. It often triggered by heat and exercise. Primary erythromelalgia occurs as an autosomal dominant mutation in the SCN9A gene which codes for a voltage-gated sodium channel involved in pain perception [20].

Secondary erythromelalgia, which occurs more commonly, has been linked to a variety of other conditions such as myeloproliferative disorders. Secondary erythromelalgia presents at an older age and with a more asymmetrical distribution compared to primary erythromelalgia. Although not usually biopsied for diagnosis, pathology can show decreased nerve density in the epidermis [20].

Patients with erythromelalgia derive relief of their symptoms by cooling the affected areas with cold water immersion to relieve the raised skin temperature; in addition, elevating the extremities may also be helpful. Sodium channel inhibitors, such as mexiletine and ranolazine, have been successful in treating some of the patients with primary erythromelalgia. In contrast, aspirin may be helpful in patients with secondary erythromelalgia related to thrombocytopenia, polycythemia, or hematologic dyscrasias. In patients with other etiologies for secondary erythromelalgia, potentially effective drugs include anticonvulsants, calcium channel blockers, serotonin reuptake inhibitors, and tricyclic antidepressants [20].

## Conclusions

Erythema ab igne is a hyperpigmented, net-like skin condition that occurs secondary to prolonged heat exposure insufficient to cause a burn. In addition to erythema ab igne, other thermal-mediated skin conditions include burns, erythromelalgia, nonmelanoma skin cancers (such as basal cell carcinoma and squamous cell carcinoma), ultraviolet radiation-associated conditions (such as beach feet and sunburns), and urticaria subtypes (such as cholinergic, localized heat, and solar). Removal of the causative agent can lead to resolution of many of these conditions; therefore, appropriate diagnosis is of paramount importance.
